# Symmetry of antiferroelectric crystals crystallized in polar point groups

**DOI:** 10.1107/S2052252522006017

**Published:** 2022-06-28

**Authors:** Pai Shan, Xifa Long

**Affiliations:** a Key Laboratory of Optoelectronic Materials Chemistry and Physics, Fujian Institute of Research on the Structure of Matter, Chinese Academy of Sciences, Fuzhou, Fujian 350002, People’s Republic of China

**Keywords:** crystal engineering, properties of solids, inorganic materials

## Abstract

Symmetry is an essential concept in physics, chemistry and materials science. Comprehensive, authoritative and accessible symmetry theory can provide a strong impetus for the development of related materials science. Through the sustained efforts of physicists and crystallographers, researchers have mastered the relationship between structural symmetry and ferroelectricity, which demands crystallization in the 10 polar point groups. However, the symmetry requirement for antiferroelectricity is still ambiguous, and polar crystals possessing antiferroelectricity seem contradictory. This work systematically and comprehensively studies the transformation of dipole moments under symmetry operations, using accessible geometric methods and group theory. The results indicate crystals that crystallize in polar point groups 2 (*C*_2_), *m* (*C*_1h_), *mm*2 (*C*_2v_), 4 (*C*_4_), 4*mm* (*C*_4v_), 3*m* (*C*_3v_), 6 (*C*_6_) and 6*mm* (*C*_6v_) also possess anti-polar structure and are capable of Kittel-type antiferroelectricity. The anti-polar direction of each point group is also highlighted, which could provide a straightforward guide for antiferroelectric property measurement. Like ferroelectric crystals, antiferroelectric crystals belonging to polar point groups have great potential to become a family of important multifunctional electroactive and optical materials. This contribution refines antiferroelectric theory, will help facilitate and stimulate the discovery and rational design of novel antiferroelectric crystals, and enrich the potential functional applications of antiferroelectric materials.

## Abstract 2

AFE crystals could also crystallize in polar point groups. If the dipole moment is reversible along the anti-polar axis under an external electric field, the polar crystal is an AFE crystal.

## 1. Introduction

Symmetry is crucial in crystallography, because symmetry is relevant to many physical and chemical crystal properties (Aroyo, 2016 ▸; Authier, 2013 ▸). In many cases, if the symmetry of a crystal or compound is known, researchers can make predictions abouts some of the important properties without having to resort to complicated calculations or experimentations (Powell, 2010 ▸). This is very useful for the discovery and design of new materials for special applications. For example, second-order nonlinear optical (NLO) response (Mutailipu *et al.*, 2021 ▸), piezoelectricity (Qiu *et al.*, 2020 ▸), as well as Pockets electro-optic (EO) effects (Gao *et al.*, 2019 ▸; Kong *et al.*, 2020 ▸) only exist in crystals that crystallize in non-centrosymmetric point groups (Halasyamani & Poeppelmeier, 1998 ▸). Meanwhile, ferroelectricity and pyroelectric properties demand that crystals crystallize in the 10 polar point groups (Halasyamani & Poeppelmeier, 1998 ▸). Comprehensive, authoritative and accessible theoretical guidance provides a strong impetus for the development of related materials science. Conversely, ambiguous symmetry will lead to inefficient development of a special functional material.

Antiferroelectric (AFE) materials can be applied in many scientific and technical fields, such as energy storage devices (Shkuratov *et al.*, 2019 ▸; Qi & Zuo, 2019 ▸; Luo *et al.*, 2019 ▸), electrocaloric cooling devices (Zhuo *et al.*, 2018 ▸; Peng *et al.*, 2013 ▸), above-bandgap photovoltages (Pérez-Tomás *et al.*, 2016 ▸) and electrostrictive devices (Qi & Zuo, 2020 ▸; Kobayashi *et al.*, 2018 ▸) *etc*. In the current period of rapid technological breakthroughs, the need for rational design and discovery of novel AFE materials is greater than ever before. However, according to *Reaxys* (latest version 2022/3/2; Goodman, 2009 ▸), there are 688 AFE materials and 9321 ferroelectric (FE) materials documented in the literature. The quantity of AFE materials is less than one-thirteenth of FE materials. It is well known that ferroelectricity demands crystallization in polar point groups. However, the symmetry requirement for antiferroelectricity is still ambiguous, and this may be the primary reason limiting the development of AFE materials science; there is an urgent need to refine the symmetry theory of AFE crystals.

The theory of AFE crystals was first proposed by Kittel (1951 ▸); in the same year, the first AFE material PbZrO_3_ was reported (Sawaguchi *et al.*, 1951 ▸). Kittel predicted that AFE phenomenon only occurs in centrosymmetric compounds or crystals without macroscopic spontaneous polarization. Crystallographers classify crystal structures into 32 different point groups or crystal classes according to their symmetry, including 11 centrosymmetric point groups and 21 non-centrosymmetric point groups. As all polar point groups belong to the non-centrosymmetric point group, according to the theory of Kittel, polar crystals cannot exhibit antiferroelectricity. Russian scientist Zheludev put forward that the Kittel-type AFE crystal can also crystallize in polar point groups (Zheludev, 1971 ▸) using the Curie and Neumann principles. However, he did not provide an explicit declaration of which polar point groups were capable of antiferroelectricity, only a table entitled ‘change in symmetry accompanying antipolarization in cubic crystals along the nonpolar direction’ was presented, which included polar point groups 2 and *mm*2. Following Zheludev’s work, Blinc and Zeks also studied the symmetry requirement for Kittel-type antiferroelectricity, they proposed that there are 27 point groups possessing antiferroelectricity, including all 10 polar point groups (Blinc & Zeks, 1974 ▸). Though Zheludev and Blinc both used the Curie and Neumann principles to study the relationship between symmetry and Kittel-type AFE crystal, the tables ‘change in symmetry accompanying antipolarization in cubic crystals along nonpolar directions’ in their respective works are different (Zheludev, 1971 ▸; Blinc & Zeks, 1974 ▸). Moreover, deeply and extensively influenced by the viewpoint of Kittel, until now chemistry and materials scientists mainly limit their search for new AFE crystals to centrosymmetric crystals (Uppuluri *et al.*, 2019 ▸; Wu *et al.*, 2019 ▸; Li *et al.*, 2017 ▸); AFE crystals or compounds crystallized in polar point group are infrequent. Recently, our group discovered a novel AFE crystal, K_3_Nb_3_B_2_O_12_, belonging to the polar point group *mm*2 (*C*_2*v*_) (Shan *et al.*, 2020 ▸), which exhibits excellent EO effects. This supports the possibility that polar crystals can also exhibit antiferroelectricity, with more potential applications than centrosymmetric AFE crystals. Hence, here we analyse the symmetry of 10 polar point groups in turn, using accessible geometric methods and group theory. We not only distinguish which polar point groups can possess anti-polar structure features, but also point out the anti-polar direction of each point group. We also discuss the unconventional phenomenon and potential applications of AFE crystals that crystallize in polar point groups.

## 2. Symmetry analysis

**Figure 1:**
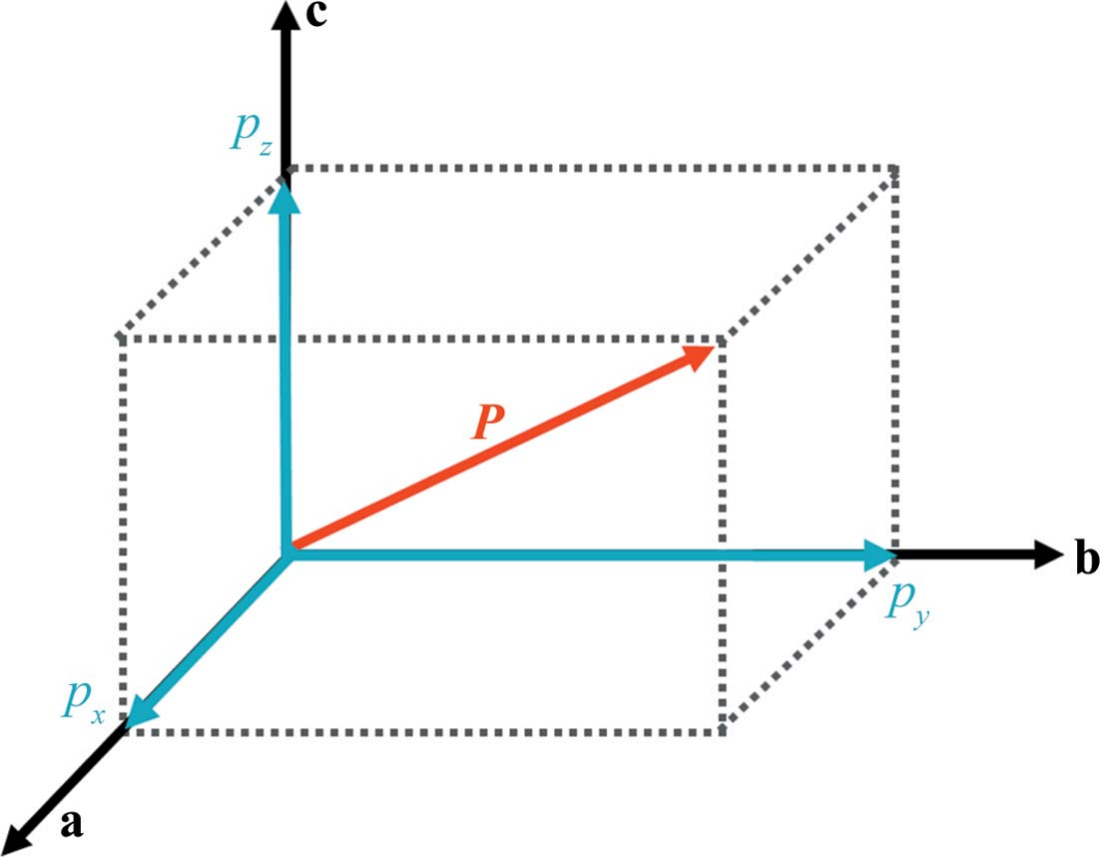
Dipole moment ***P*** is a vector. In a crystallographic coordinate system, it can be expressed as ***P*** = *p_x_* **i** + *p_y_* **j** + *p_z_* **k**. Where **i**, **j**, **k** are the unit vectors, *p_x_*, *p_y_* and *p_z_* represent the dipole moment components along crystallographic *a*, *b* and *c* axes, respectively.

A Kittel-type AFE crystal is defined as a crystal with an antiparallel arrangement of electric dipoles at adjacent crystalline sublattices, and the antiparallel electric dipoles can be reversible (switchable) by external electric field. Therefore, the key analysis of the symmetry requirement for an AFE crystal is the study of the transformation of the dipole moment under symmetry operations. The dipole moment ***P*** is a vector, in a crystallographic coordinate system it can be expressed as

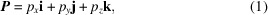

where **i**, **j** and **k** are the unit vectors; *p_x_*, *p_y_* and *p_z_* represent the dipole moment components along the *a*, *b* and *c* crystallographic axes, respectively (Fig. 1 ▸). In this paper, the crystallographic coordinate system is in accordance with the *International Tables for Crystallography* (Powell, 2010 ▸; Aroyo, 2016 ▸).

Neither the magnitude nor the direction of a dipole moment change under a translation operation; therefore, we only need to discuss 32 point groups instead of 230 space groups. Among the 32 crystallographic point groups, there are 10 polar point groups: 1 (*C*_1_), 2 (*C*_2_), *m* (*C*_1*h*_ = *C*_*s*_), *mm*2 (*C*_2*v*_), 4 (*C*_4_), 4*mm* (*C*_4*v*_), 3 (*C*_3_), 3*m* (*C*_3*v*_), 6 (*C*_6_) and 6*mm* (*C*_6*v*_). Here, the International symbols and Schoenflies symbols are given in brackets. In this section, we analyse the symmetry of 10 polar point groups from low to high symmetry, and distinguish which polar point groups can possess anti-polar structure features using an accessible mathematical method.

### 2.1. Point groups 1 (*C*_1_) and 3 (*C*_3_)

We first study point group 1 (*C*_1_), which possesses the lowest symmetry among the 32 crystallographic point groups. There is only one identity symmetry operation for 1 (*C*_1_). A crystal with an anti-polar structure implies there is a higher symmetry than identity symmetry operation 1. Moreover, in point group 1 (*C*_1_), there is no nonpolar direction (Aroyo, 2016 ▸). Hence, we have a different opinion to Blinc and Zeks: point group 1 (*C*_1_) is not suitable for Kittel-type antiferroelectricity.

**Figure 2:**
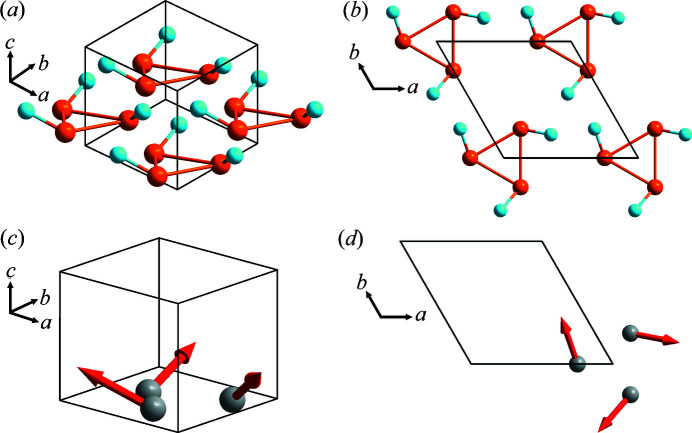
(*a*) and (*b*) Schematic for point group 3 (*C*_3_) viewed along different crystallographic directions. (*c*) and (*d*) Transformation of the dipole moment in point group 3 (*C*_3_) viewed along different crystallographic directions. In (*a*)–(*d*), *a* = *b* ≠ *c*, α = β = 90°, γ = 120°.

Point group 3 (*C*_3_) contains only identity symmetry element 1 and a threefold rotation axis. Suppose there is a motif A with the dipole moment ***P***_A_ (*p_x_*, *p_y_*, *p_z_*), two new motifs B and C are generated with dipole moments ***P***_B_ (−*p_y_*, *p_x_* − *p_y_*, *p_z_*) and ***P***_C_ (*p_y_ − p_x_*, −*p_x_*, *p_z_*) under symmetry operation 3 (Fig. 2 ▸). Note that, in trigonal and hexagonal systems discussed here, the angle between the *a* axis and the *b* axis is 120°, and the *c* axis is perpendicular to the *ab* plane. The motif can be a molecule or a building block of a network structure *etc*. In point group 3 (*C*_3_), the threefold rotation axis (*c* axis) is the polar axis, and the arbitrarily oriented direction [*hk*0] lying in the plane perpendicular to threefold rotation axis (*c* axis) is the nonpolar direction. However, there is no anti-polar direction, as we could not separate odd motifs into two uniform groups. If a crystal is anti-polar, it must be nonpolar, but the converse is not true. Blinc and Zeks considered all nonpolar directions as anti-polar directions, then classified 3 (*C*_3_) into the Kittel-type AFE point group. To date, there is no report of AFE crystals or compounds belonging to point groups 1 (*C*_1_) or 3 (*C*_3_) in the literature.

### 2.2. Point groups *m* (*C*_1*h*_) and 2 (*C*_2_)

We now consider point groups *m* (*C*_1*h*_) and 2 (*C*_2_). Beyond identity symmetry element 1, each contains only one other symmetry element, a mirror plane *m* and a twofold rotation axis, respectively.

**Figure 3:**
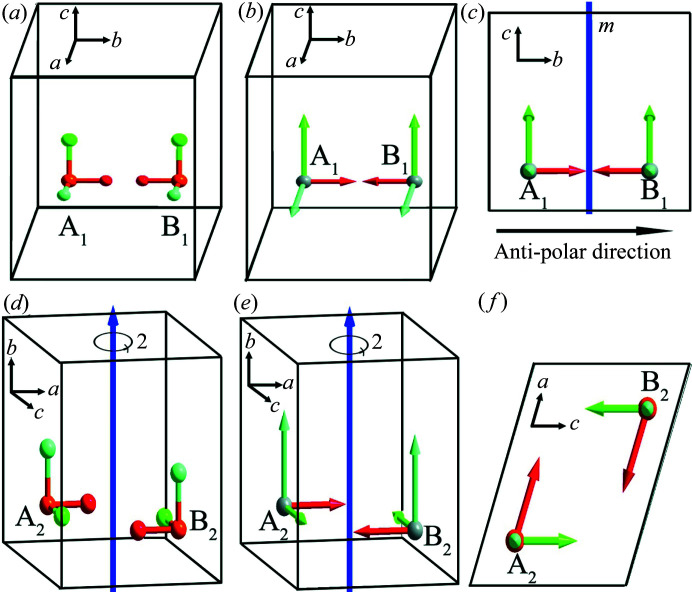
(*a*) Schematic for point group *m* (*C*_1*h*_). (*b*) and (*c*) Transformation of the dipole moment in point group *m* (*C*_1*h*_) viewed along different crystallographic directions. (*d*) Schematic for point group 2 (*C*_2_). (*e*) and (*f*) Transformation of the dipole moment in point group 2 (*C*_2_) viewed along different crystallographic directions. In (*a*)–(*f*), *a* ≠ *b* ≠ *c*, α = γ = 90°, β ≠ 90°.

Suppose there is a motif A_1_ with the dipole moment ***P***_A1_ (*p_x_*, *p_y_*, *p_z_*) in a crystal in point group *m* (*C*_1*h*_). Under symmetry operation *m*, a new motif B_1_ is generated, and the dipole moment of motif B_1_ must be ***P***_B1_ (*p_x_*, −*p_y_*, *p_z_*), as shown in Figs. 3 ▸(*a*)–3(*c*). Obviously, it is easy to divide a crystal in point group *m* (*C*_1*h*_) into two sublattices by the mirror plane *m* [Fig. 3 ▸(*c*)], with antiparallel dipoles along the crystallographic *b* axis (here the *b* axis is perpendicular to *m*). Hence, we call the *b* axis the ‘anti-polar axis’ of *m* (*C*_1*h*_), *i.e.* the [010] direction is the ‘anti-polar direction’ of *m* (*C*_1*h*_). All directions except [010] are polar in point group *m* (*C*_1*h*_). In practice, to obtain the perfect double polarization versus electric field (*P*–*E*) hysteresis loop of an AFE crystal in point group *m* (*C*_1h_), the optimum external electric field direction should apply along the [010] direction.

Similarly, suppose there is a motif A_2_ with the dipole moment ***P***_A2_ (*p_x_*, *p_y_*, *p_z_*) in a crystal in point group 2 (*C*_2_). Under symmetry operation 2, a new motif B_2_ is generated, and the dipole moment of motif B_2_ must be ***P***_B2_ (−*p_x_*, *p_y_*, −*p_z_*), as shown in Figs. 3 ▸(*d*)–3(*f*). Obviously, the *b* axis is the polar axis of point group 2 (*C*_2_), here, the *b* axis is parallel to the twofold rotation axis [Figs. 3 ▸(*d*) and 3 ▸(*e*)]. The arbitrarily oriented direction [*h*0*l*] lying in the plane perpendicular to the twofold rotation axis (*b* axis) is the anti-polar direction of point group 2 (*C*_2_) [Fig. 3 ▸(*f*)]. The arbitrary plane parallel to the twofold rotation axis (*b* axis) can divide a crystal in point group 2 (*C*_2_) into two sublattices with antiparallel dipoles, *i.e.* point group 2 (*C*_2_) also permits Kittel-type antiferroelectricity.

### 2.3. Point groups 4 (*C*_4_) and 6 (*C*_6_)

Both point groups 4 (*C*_4_) and 6 (*C*_6_) contain a symmetry element twofold rotation axis. In group theory, point group 2 (*C*_2_) is the subgroup of point groups 4 (*C*_4_) and 6 (*C*_6_) (Fig. S1 of the supporting information). Hence, crystals in point groups 4 (*C*_4_) or 6 (*C*_6_) also permit antiferroelectricity.

**Figure 4:**
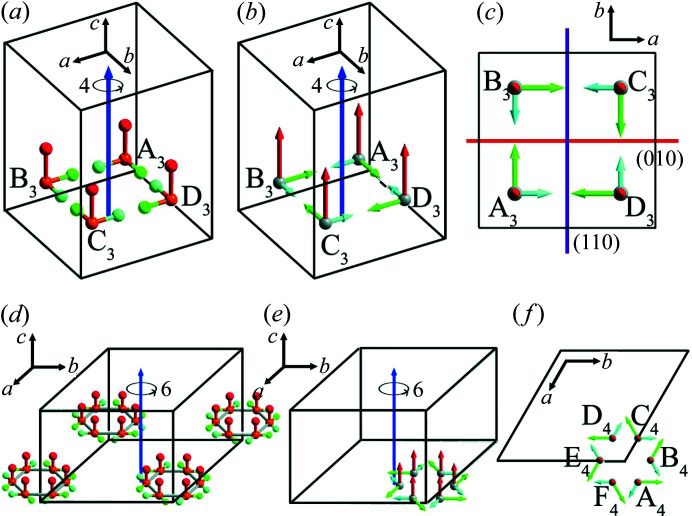
(*a*) Schematic for point group 4 (*C*_4_). (*b*) and (*c*) Transformation of the dipole moment in point group 4 (*C*_4_) viewed along different crystallographic directions. In (*a*)–(*c*), *a* = *b* ≠ *c*, α = β = γ = 90°. (*d*) Schematic for point group 6 (*C*_6_). (*e*) and (*f*) Transformation of the dipole moment in point group 6 (*C*_6_) viewed along different crystallographic directions. In (*d*)–(*f*), *a* = *b* ≠ *c*, α = β = 90°, γ = 120°.

The same conclusion could also be obtained by analysis of the transformation of the dipole moment under symmetry operations. Suppose there is a motif A_3_ with the dipole moment ***P***_A3_ (*p_x_*, *p_y_*, *p_z_*) in a crystal in point group 4 (*C*_4_). Three new motifs B_3_, C_3_ and D_3_ are generated with dipole moments ***P***_B3_ (*p_y_*, −*p_x_*, *p_z_*), ***P***_C3_ (−*p_x_*, −*p_y_*, *p_z_*) and ***P***_D3_ (−*p_y_*, *p_x_*, *p_z_*) under a fourfold rotation symmetry operation [Figs. 4 ▸(*a*)–4(*c*)]. Similarly, if there is a motif A_4_ with the dipole moment ***P***_A4_ (*p_x_*, *p_y_*, *p_z_*) in a crystal in point group 6 (*C*_6_), then 5 new motifs B_4_, C_4_, D_4_, E_4_ and F_4_ are generated with dipole moments ***P***_B4_ (*p_x_* − *p_y_*, *p_x_*, *p_z_*), ***P***_C4_ (−*p_y_*, *p_x_* − *p_y_*, *p_z_*), ***P***_D4_ (−*p_x_*, −*p_y_*, *p_z_*), ***P***_E4_ (−*p_x_* + *p_y_*, −*p_x_*, *p_z_*) and ***P***_F4_ (*p_y_*, −*p_x_* + *p_y_*, *p_z_*) under a sixfold rotation symmetry operation [Figs. 4 ▸(*d*)–4(*f*)]. In the hexagonal system, the angle between the *a* axis and the *b* axis is 120°, and the *c* axis is perpendicular to the *ab* plane. The situations of point groups 4 (*C*_4_) and 6 (*C*_6_) are similar to 2 (*C*_2_), the fourfold or sixfold rotation axis (*c* axis) is the polar axis and an arbitrarily oriented direction [*hk*0] lying in the plane perpendicular to the *c* axis is the anti-polar direction. The arbitrary plane parallel to the *c* axis can divide crystals in point groups 4 (*C*_4_) or 6 (*C*_6_) into two sublattices with antiparallel dipoles.

Here we take 4 (*C*_4_) as an example: in Fig. 4 ▸(*c*), the (100) plane divides the crystal lattice into two sublattices, the left sublattice contains motifs A_3_ and B_3_ with a net dipole moment (*p_x_* + *p_y_*, *p_y_*−*p_x_*, 2*p_z_*), and the right sublattice contains motifs C_3_ and D_3_ with a net dipole moment (−*p_x_* − *p_y_*, *p_x_* − *p_y_*, 2*p_z_*). In the left and right sublattices, the magnitude of the dipole moments and their components along the three crystallographic axes are equal. However, the direction of the dipole moment in two sublattices is the opposite in the (001) plane. As another example, the (010) plane also divides the crystal lattice into two sublattices [Fig. 4 ▸(*c*)], the bottom sublattice contains motifs A_3_ and D_3_ with a net dipole moment (*p_x_* − *p_y_*, *p_x_* + *p_y_*, 2*p_z_*), and the top sublattice contains motifs B_3_ and C_3_ with a net dipole moment (*p_y_* − *p_x_*, −*p_x_* − *p_y_*, 2*p_z_*). Obviously, the dipole moments in the top and bottom sublattices are also in an antiparallel arrangement in the (001) plane. Hence, point groups 4 (*C*_4_) and 6 (*C*_6_) both permit antiferroelectricity.

### 2.4. Point groups *mm*2 (*C*_2*v*_), 4*mm* (*C*_4*v*_), 6*mm* (*C*_6*v*_) and 3*m* (*C*_3*v*_)

Point groups *mm*2 (*C*_2*v*_), 3*m* (*C*_3*v*_), 4*mm* (*C*_4*v*_) and 6*mm* (*C*_6*v*_) have a two-, three-, four- or sixfold rotation axis, plus 2, 3, 4 and 6 mirror planes, respectively. In group theory, both 2 (*C*_2_) and *m* (*C*_1 h_) are the subgroups of point groups *mm*2 (*C*_2*v*_), 4*mm* (*C*_4*v*_) and 6*mm* (*C*_6*v*_) (Fig. S1). As all mirror planes are parallel to their rotation axes, the two-, four- or sixfold rotation axis (all are the *c* axis) is the polar axis of *mm*2 (*C*_2*v*_), 4*mm* (*C*_4*v*_) and 6*mm* (*C*_6*v*_), respectively. The arbitrarily oriented direction [*hk*0] lying in the plane perpendicular to the two-, four- or sixfold rotation axis (*c* axis) is the anti-polar direction of *mm*2 (*C*_2*v*_), 4*mm* (*C*_4*v*_) and 6*mm* (*C*_6*v*_), respectively. The arbitrary plane parallel to the *c* axis can divide a crystal in point groups *mm*2 (*C*_2*v*_), 4*mm* (*C*_4*v*_) and 6*mm* (*C*_6*v*_) into two sublattices with antiparallel dipoles and hence permit antiferroelectricity.

**Figure 5:**
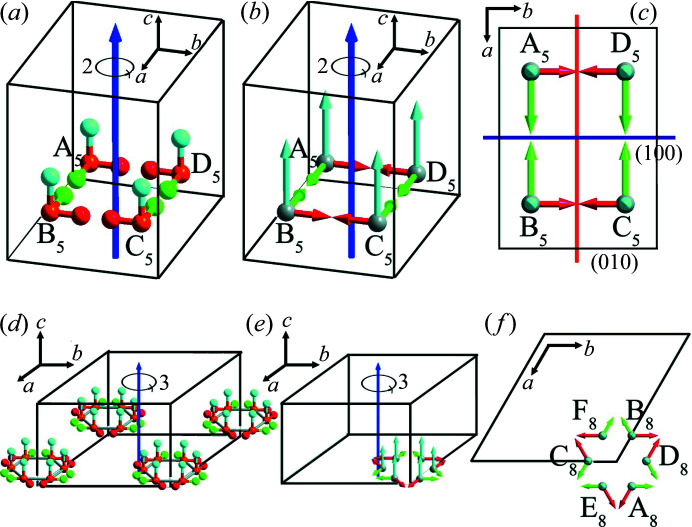
(*a*) Schematic for point group *mm*2 (*C*_2*v*_). (*b*) and (*c*) Transformation of the dipole moment in point group *mm*2 (*C*_2*v*_) viewed along different crystallographic directions. In (*a*)–(*c*), *a* ≠ *b* ≠ *c*, α = β = γ = 90°. (*d*) Schematic for point group 3*m* (*C*_3*v*_). (*e*) and (*f*) Transformation of the dipole moment in point group 3*m* (*C*_3*v*_) viewed along different crystallographic directions. In (*d*)–(*f*), *a* = *b* ≠ *c*, α = β = 90°, γ = 120°.

We could also employ the same mathematical method employed in the above sections to analyse the symmetry of point groups *mm*2 (*C*_2*v*_), 4*mm* (*C*_4*v*_) and 6*mm* (*C*_6*v*_). Suppose there is a motif A with the dipole moment ***P***_A_ (*p_x_*, *p_y_*, *p_z_*) in the above three point groups. In a crystal in point group *mm*2 (*C*_2*v*_), three new motifs are generated with dipole moments ***P***_B5_ (−*p_x_*, *p_y_*, *p_z_*), ***P***_C5_ (−*p_x_*, −*p_y_*, *p_z_*) and ***P***_D5_ (*p_x_*, −*p_y_*, *p_z_*) [Figs. 5 ▸(*a*)–5(*c*)]. In a crystal in point group 4*mm* (*C*_4*v*_), seven new motifs are generated with dipole moments ***P***_B6_ (−*p_y_*, *p_x_*, *p_z_*), ***P***_C6_ (−*p_x_*, −*p_y_*, *p_z_*), ***P***_D6_ (*p_y_*, −*p_x_*, *p_z_*), ***P***_E6_ (*p_x_*, −*p_y_*, *p_z_*), ***P***_F6_ (−*p_y_*, −*p_x_*, *p_z_*), ***P***_G6_ (−*p_x_*, *p_y_*, *p_z_*) and ***P***_H6_ (*p_y_*, *p_x_*, *p_z_*). In a crystal in point group 6*mm* (*C*_6*v*_), eleven new motifs are generated with dipole moments ***P***_B7_ (*p_x_* − *p_y_*, *p_x_*, *p_z_*), ***P***_C7_ (−*p_y_*, *p_x_* − *p_y_*, *p_z_*), ***P***_D7_ (−*p_x_*, −*p_y_*, *p_z_*), ***P***_E7_ (*p_y_* − *p_x_*, −*p_x_*, *p_z_*), ***P***_F7_ (*p_y_*, *p_y_* − *p_x_*, *p_z_*), ***P***_G7_ (*p_x_* − *p_y_*, −*p_y_*, *p_z_*), ***P***_H7_ (−*p_y_*, −*p_x_*, *p_z_*), ***P***_I7_ (−*p_x_*, *p_y_* − *p_x_*, *p_z_*), ***P***_J7_ (*p_y_* − *p_x_*, *p_y_*, *p_z_*), ***P***_K7_ (*p_y_*, *p_x_*, *p_z_*) and ***P***_L7_ (*p_x_*, *p_x_* − *p_y_*, *p_z_*). We can use a similar method to that described in the above sections to divide the above crystal lattice into two sublattices with antiparallel dipoles.

Here we take *mm*2 (*C*_2*v*_) as an example [Figs. 5 ▸(*a*)–5(*c*)]. In Fig. 5 ▸(*c*), the (100) plane divides the crystal lattice into two sublattices, the top sublattice contains motifs A_5_ and D_5_ with a net dipole moment (2*p_x_*, 0, 2*p_z_*), and the bottom sublattice contains motifs B_5_ and C_5_ with a net dipole moment (−2*p_x_*, 0, 2*p_z_*). Obviously, the magnitudes of the dipole moment components are equal and the direction of the dipole moment in the two sublattices is the opposite in the (001) plane. In other words, the dipole moments in the top and bottom sublattices are in an antiparallel arrangement in the (001) plane. The (010) plane also divides the crystal lattice into two sublattices [Fig. 5 ▸(*c*)], the left sublattice contains motifs A_5_ and B_5_ with a net dipole moment (0, 2*p_y_*, 2*p_z_*), and the right sublattice contains motifs C_5_ and D_5_ with a net dipole moment (0, −2*p_y_*, 2*p_z_*). The dipole moments in the left and right sublattices are also in antiparallel arrangement in the (001) plane. The varied mathematical division methods imply anisotropic properties of the crystal. When an electric field is applied on different crystal orientations, the shape of the double *P–E* hysteresis loop may exhibit differences, for example different maximum polarization (*P*_max_).

Point group 3*m* (*C*_3*v*_) is different, it only contains the mirror plane *m* and a threefold rotation axis, without a twofold rotation axis. Suppose there is a motif A_8_ with the dipole moment ***P***_A8_ (*p_x_*, *p_y_*, *p_z_*) in a crystal in point group 3*m* (*C*_3*v*_). Five new motifs B_8_, C_8_, D_8_, E_8_ and F_8_ are generated with dipole moments ***P***_B8_ (−*p_y_*, *p_x_* − *p_y_*, *p_z_*), ***P***_C8_ (−*p_x_* + *p_y_*, −*p_x_*, *p_z_*), ***P***_D8_ (−*p_x_ + p_y_*, *p_y_*, *p_z_*), ***P***_E8_ (*p_x_*, *p_x_* − *p_y_*, *p_z_*) and ***P***_F8_ (−*p_y_*, −*p_x_*, *p_z_*). The threefold rotation axis (*c* axis) is still the polar axis of point group 3*m* (*C*_3*v*_), and there are only three anti-polar directions, which are perpendicular to each mirror plane *m*. Three anti-polar directions are also perpendicular to the threefold rotation axis. A crystal in point group 3*m* (*C*_3*v*_) can be divided into two sublattices with antiparallel dipoles by each mirror plane *m*, and hence permits antiferroelectricity.

Our analysis process is shown in Fig. S1. A brief discussion of nonpolar point groups is also presented in the supporting information.

## 3. Results and discussion

According to the symmetry analysis in Section 2, eight polar point groups including 2 (*C*_2_), *m* (*C*_1*h*_), *mm*2 (*C*_2*v*_), 4 (*C*_4_), 4*mm* (*C*_4*v*_), 3*m* (*C*_3v_), 6 (*C*_6_) and 6*mm* (*C*_6*v*_) possess an anti-polar direction, and are capable of antiferroelectricity. For *m* (*C*_1*h*_), the anti-polar direction is perpendicular to the mirror plane *m*. For 3*m* (*C*_3*v*_), there are three anti-polar directions perpendicular to three respective mirror planes. For point groups 2 (*C*_2_), *mm*2 (*C*_2*v*_), 4 (*C*_4_), 4*mm* (*C*_4*v*_), 6 (*C*_6_) and 6*mm* (*C*_6*v*_), the arbitrarily oriented direction [*h*0*l*] or [*hk*0] lying in the plane perpendicular to the two-, four-, and sixfold rotation axes is their anti-polar direction. The common characteristic of these polar point groups is that the anti-polar axis is perpendicular to a special polar axis in the crystal.

**Figure 6:**
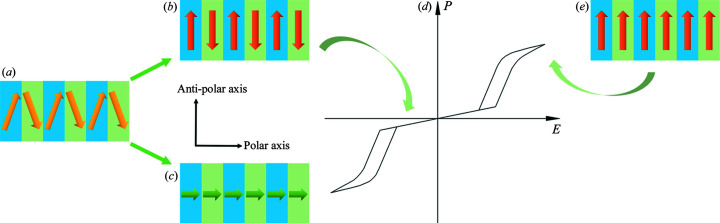
(*a*) Schematic of dipole moments distribution in an AFE crystal crystallized in a polar point group. (*b*) Dipole moment components are in an antiparallel arrangement along the anti-polar axis. (*c*) Dipole moment components are in a parallel arrangement along the polar axis. (*d*) Schematic of double *P*–*E* hysteresis loops of an AFE crystal. (*e*) Dipole moments form a parallel arrangement in the field-induced FE phase.

The dipole moments distribution in an AFE crystal crystallized in a polar point group can be described by Fig. 6 ▸(*a*). Dipole moment components are in an antiparallel arrangement along the anti-polar axis [Fig. 6 ▸(*b*)], and in a parallel arrangement along the polar axis [Fig. 6 ▸(*c*)]. If the dipole moment is reversible along the polar axis under an external electric field, the polar crystal is an FE crystal. If the dipole moment is reversible along the anti-polar axis under an external electric field, the polar crystal is an AFE crystal. If we want to obtain the double *P–E* hysteresis loop [Fig. 6 ▸(*d*)] of a polar AFE crystal, the external electric field should be applied parallel to the anti-polar direction, and the dipole moments will become a parallel arrangement in the field-induced FE phase [Fig. 6 ▸(*e*)]. If the applied electric field is accurate along the anti-polar direction, remnant polarization *P*_r_ of double *P–E* hysteresis loops will be invariably zero, which is consistent with the traditional knowledge of AFE crystals.

It is becoming apparent that the centrosymmetric criterion is insufficient to distinguish whether a crystal can produce antiferroelectricity. Instead, whether a crystal or compound contains an anti-polar axis or an anti-polar direction should be the new criteria. However, an anti-polar structure is the necessary but not insufficient condition for AFE crystals, just like the relationship between polar structures and FE crystals. FE or AFE crystals also demand the dipole moment is reversible by an external electric field. The double *P–E* hysteresis loops are still the most important features of an AFE crystal crystallized in polar point groups, corresponding to the field-induced AFE-to-FE phase transition. Compared with the work of Zheludev, Blinc and others, our work not only highlights which polar point groups are capable of antiferroelectricity, but also identifies the anti-polar direction of each point group in a systematic, comprehensive and accessible manner.

Some unconventional phenomena may occur in AFE crystals that crystallize in polar point groups. First, these AFE crystals may possess macroscopic spontaneous polarization along their polar axis [Fig. 6 ▸(*c*)]. However, the spontaneous polarization is zero along their anti-polar axis [Fig. 6 ▸(*b*)], as the crystals possess anisotropy. Second, AFE crystals can exhibit pyroelectric properties, piezoelectricity, NLO response and Pockets EO effect, as all polar point groups belong to the non-centrosymmetric point group. Some AFE crystals that crystallize in non-centrosymmetric point groups have been reported, such as NH_4_H_2_PO_4_ [*P*2_1_2_1_2_1_ (Lasave *et al.*, 2007 ▸; Hewat, 1973 ▸)] and NH_4_H_2_AsO_4_ [*P*2_1_2_1_2_1_ (Fu *et al.*, 2015 ▸)]. However, the AFE phase only exists at extremely low temperature for NH_4_H_2_PO_4_ [below 148 K (Choudhury *et al.*, 2014 ▸)] and NH_4_H_2_AsO_4_ [below 215 K (Fu *et al.*, 2015 ▸)], the low temperature limits the measurement of the above-mentioned properties of the AFE phase. Recently, the second-order NLO response and Pockets EO effect have been discovered in the polar AFE crystal K_3_Nb_3_B_2_O_12_ (Shan *et al.*, 2020 ▸, 2021 ▸). AFE crystals have great potential to be applied as novel EO materials with superior performance because of the considerable ionic contribution. Third, there may still be a great number of hidden AFE crystals yet to be discovered in non-centrosymmetric point groups.

AFE compounds can form various solid materials, the above symmetry analysis is not only applicable to these crystals, but also suitable for other AFE solid materials such as ceramics and thin films *etc*.

## 4. Conclusions

Our work not only identifies which polar point groups are capable of AFE, but also highlights the anti-polar direction of each point group, which could provide a straightforward guide for AFE property measurement. Among the 10 polar point groups, there are 8 point groups that possess anti-polar direction which are capable of Kittel-type antiferroelectricity. This work provides an authoritative and accessible theoretical direction for discovery and rational design of new AFE crystals.

This work may subvert some traditional knowledge of AFE science. We argue that centrosymmetry is no longer a suitable criterion to distinguish whether a crystal is capable of antiferroelectricity. Instead, the presence of an anti-polar axis or anti-polar direction should be the new criteria. AFE crystals can also possess spontaneous polarization, and exhibit pyroelectric properties, piezoelectricity, NLO response and Pockets EO effect. Like FEs, AFEs have great potential to become a family of important multifunctional electroactive and optical materials.

## Supplementary Material

Supporting figures and discussion. DOI: 10.1107/S2052252522006017/zx5026sup1.pdf
