# Supplementary material for: Symmetry of antiferroelectric crystals crystallized in polar point groups
Source: IUCrJ. 2022 Jun 28;9(Pt 4):516–22. doi: 10.1107/S2052252522006017 (PMC9252155; doi:10.1107/S2052252522006017)
Supplement: Supplementary file 1 [file m-09-00516-sup1.pdf]

IUCrJ

Volume 9 (2022)

Supporting information for article:

Symmetry of antiferroelectric crystals crystallized in polar point groups

Pai Shan and Xifa Long

Symmetry of antiferroelectric crystals crystallizing in polar point groups

Pai Shan and Xifa Long*

Key Laboratory of Optoelectronic Materials Chemistry and Physics, Fujian Institute of Research on the Structure of Matter, Chinese Academy of Sciences, Fuzhou, Fujian 350002, China

I. The logicality of our analysis process.

We analyze the relationship between crystal symmetry and Kittel-type antiferroelectricity from low to high symmetry point groups. This is opposite to R. Blinc *et al*., they analyze the same question from high to low symmetry point groups. The logicality of our analysis process shows in Figure S1. 1 (C_1) possess the lowest symmetry, it is the subgroup of 2 (C_2), m ($C_{1h} = C_s$) and 3 (C_3), *i.e.* $1 \subseteq 2$, $1 \subseteq m$ and $1 \subseteq 3$. Here, only 2 and m possess anti-polar structure. As 2 (C_2) is the subgroup of 4 (C_4) and 6 (C_6), *i.e.* $2 \subseteq 4$ and $2 \subseteq 6$, hence 4 (C_4) and 6 (C_6) also possess anti-polar structure. Point group m is the subgroup of $3m$ (C_{3v}), *i.e.* $m \subseteq 3m$, hence $3m$ (C_{3v}) possesses anti-polar structure. Point groups $mm2$ (C_{2v}), $4mm$ (C_{4v}) and $6mm$ (C_{6v}) simultaneously contain subgroups 2 (C_2) and m ($C_{1h} = C_s$), *i.e.* $2 \subseteq mm2$, $2 \subseteq 4mm$ and $2 \subseteq 6mm$, $m \subseteq mm2$, $m \subseteq 4mm$ and $m \subseteq 6mm$, hence $mm2$ (C_{2v}), $4mm$ (C_{4v}) and $6mm$ (C_{6v}) also possess anti-polar structure.

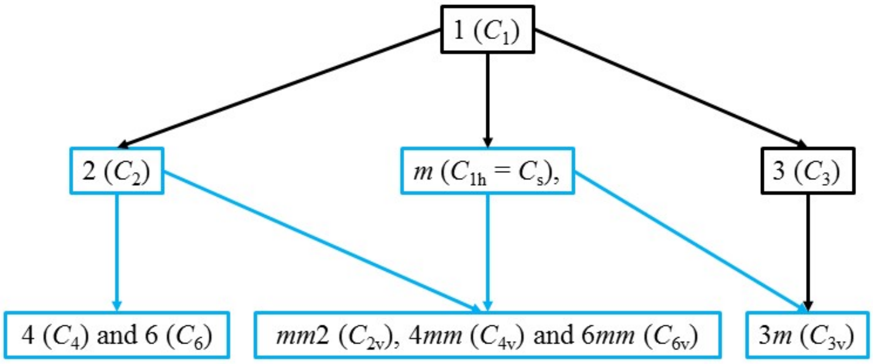

Figure S1 The relationship of 10 polar point groups. Point groups in blue boxes possess anti-polar structure. Arrows represent subgroup relationship.

II. Brief discussion of nonpolar point groups

Among 32 crystallography point groups, there are 10 polar point group and 22 nonpolar point groups. If a crystal is nonpolar, there generally are two situations: (a) Crystal constitutes of nonpolar motifs, *i.e.* motifs with zero dipole moments. Geometrically, nonpolar motifs generally possess regular geometrical configuration (Fig. S2). Such as, linear [XY_2] type, regular trigonal planar geometry, regular tetrahedron, regular hexagon, cube, regular octahedron, and so on. If a crystal constitutes of nonpolar motifs, it generally doesn't have antiferroelectricity, however, it could be the paraelectric phase of FE or AFE crystal. However, there are still some special situations. $BaTiO_3$ could exhibit double *P–E* hysteresis loops slightly above its Curie temperature. (W. J. Merz, Phys. Rev.,1953, 91, 513). (b) Crystal constitutes of polar motifs and dipole moments of motifs are antiparallel arrangement, leading to net dipole moment is zero (nonpolar). This type of crystal could possess antiferroelectricity. However, we could not explicitly know whether a crystal is paraelectric phase or AFE phase just by point group without chemical structure information. In nonpolar point groups, the threefold rotation axis won't exist alone, it is always coexistence with other symmetry operations. So, we didn't need to discuss this special case here.

Among 22 nonpolar point groups, there are 11 centrosymmetric point groups, and 11 non-centrosymmetric and nonpolar (NCNP) point groups. With respect to antiferroelectricity, NCNP point groups are equivalent to centrosymmetric point groups. This conclusion has a strong practicability. From the performance perspective, one of the most exciting aspects of AFE materials is their potential to be applied as novel energy storage materials, which is generally processed into ceramic. Compared with crystal, the anisotropy of AFE compounds will be homogenization in ceramic sample, dipole moments antiparallel arrangement in all three crystallographic

axes is beneficial. This means searching for high-energy storage AFE materials is not needed to limit to centrosymmetric compounds, but also NCNP compounds.

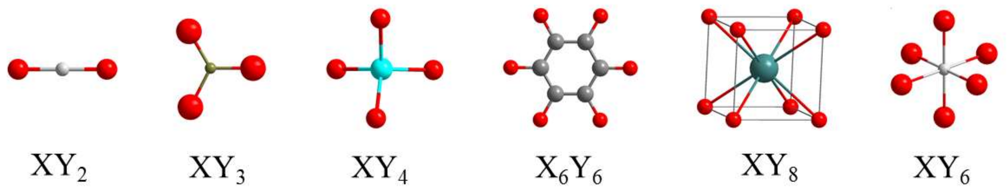

Figure S2 Schematic for nonpolar motifs with regular geometrical configuration. Linear [XY_2] type, regular trigonal planar geometry, regular tetrahedron, regular hexagon, cube and regular octahedron motifs.
